# Coexisting Atlantic Cod Ecotypes in the Barents Sea: An Issue for Managing Fisheries

**DOI:** 10.1111/eva.70250

**Published:** 2026-05-10

**Authors:** Torild Johansen, Per Erik Jorde, Jon‐Ivar Westgaard, Kevin A. Glover, Johanna Fall, Geir Dahle, Jane Godiksen

**Affiliations:** ^1^ Institute of Marine Research (IMR) Tromsø Norway; ^2^ Institute of Marine Research (IMR) Arendal Norway; ^3^ Institute of Marine Research (IMR) Bergen Norway

**Keywords:** *Gadus morhua* L, hybrids, monitoring, otoliths, stock origin

## Abstract

The largest remaining cod stock in the Atlantic, the Northeast Arctic cod (NEAC), is facing climate change and poor recruitment. It is currently assumed to represent a single biological population. In coastal waters, both in Northern Norway and down to mid‐Norway during the spawning season, NEAC overlaps with another cod ecotype, the conspecific coastal cod (CC), which is managed as a separate stock. Here, we analysed otoliths and conducted genotyping of 3900 cod to investigate population structure and potential ecotype mixtures in the Barents Sea. The annual surveys cover both the spawning and feeding areas of the NEAC stock with the purpose of collecting data for the annual stock assessment. While classification of individual cod to population‐of‐origin with the genetic markers currently available remains challenging, we—for the first time—infer the presence of CC and potential NEAC × CC hybrids also far offshore in the Barents Sea. Moreover, the CC were not homogenously distributed in the Barents Sea, but were higher in the west (including Svalbard) and in the far east, as well as near the Norwegian coast. Most of these putatively identified CC fish carried NEAC‐type otoliths. Thus, cod otolith type seems a poor indicator of genetic ancestry and instead reflects environmental conditions. Many questions remain on the temporal stability of the NEAC and CC mixing in the Barents Sea, and not least, the management implications of such stock mixing. More generally, further studies are needed on the connection between within‐species genetic differences and the implications of such differences for stock dynamics in a management context.

## 1 Introduction

In fishery management, accurate stock assessment requires that the biological populations are correctly identified and defined. Most of the statistical assessment models define stocks by the ecological paradigm that they are demographically divided (Waples and Gaggiotti 2008), implying that a stock is made up of one population, but in practical applications the stock definition is often based on national borders or jurisdictions (Saha et al. 2015). The evolutionary paradigm instead defines stocks from reproductive isolated populations where the stocks are genetically defined spawning populations but could be mechanically mixed outside spawning season.

Genetic methods have been available for fisheries management for almost 60 years (Utter 1991; Allendorf 2017) and although genetic methods are used in the monitoring of some marine stocks (Smith et al. 2005; Johansen et al. 2018; Dahle, Johansen, et al. 2018; Andersen et al. 2024), only a few studies have shown how the traditional statistical assessment models more directly can benefit from including genetic assignment for a better understanding of stock components (Spies et al. 2015; Kerr et al. 2014; Christensen et al. 2022). Within their model, Kerr et al. (2014, 2016) observed different contributions over years of three genetic components of Atlantic cod within the Gulf of Maine. In a situation where species distributions are changing, for example, due to climate change, it is particularly important to understand the population genetic structure of harvested species to avoid overexploiting vulnerable components and thereby risk losing genetic variation (Therkildsen et al. 2013; Christensen et al. 2022; Spotowitz et al. 2022).

Atlantic cod ( *Gadus morhua* L) is an economically important demersal species distributed and supporting important fisheries across the north Atlantic. Cod stocks are often composed of more than one biological population and, as such, provide challenges to management. For example, based on genetic differences, three putative populations have been inferred within Atlantic cod in the North Sea (Hutchinson et al. 2001), two in the Baltic Sea (Hemmer‐Hansen et al. 2018), two in the Celtic/Irish sea (Johansen et al. 2020), as well as multiple coastal and offshore populations of various levels of genetic differentiation found across the North Atlantic from Canada, Greenland, Iceland, and Norway (Berg et al. 2017; Therkildsen et al. 2013; Christensen et al. 2022). As many of the populations spawn in coastal waters, focus has been on defining management‐relevant population differentiation in coastal areas (Barth et al. 2017; Pampoulie et al. 2012; Sodeland et al. 2016; Knutsen et al. 2018; Johansen et al. 2018, 2020; Jorde et al. 2018, 2021).

The major cod stock in the Northeast Atlantic is the Northeast Arctic cod (hereafter: NEAC), currently assumed to represent a single biological population. NEAC has shown poor recruitment the last decade, which is also reflected in the reduction of the annual quotas (Howell et al. 2025). This stock has its nursery and feeding areas in the Barents Sea, migrating to the coast of Norway to spawn, while egg and larvae drift back with ocean currents to the Barents Sea (Vikebø et al. 2005; Olsen et al. 2010). On the spawning grounds along the Norwegian coast north of 62° N, NEAC is fished in a mixed‐stock fishery along with the more stationary coastal cod (CC). CC consists of an unknown number of populations that display a stepping‐stone genetic pattern along the Norwegian coastline (Dahle, Quintela, et al. 2018; Jorde et al. 2021; Breistein et al. 2022). For management purposes, CC has been divided into three stocks, separated at 62° and 67° N (ICES 2021). The status of the Norwegian CC stocks generally improves from south to north, though the fishing pressure north of 67° N, where most of the mixed fishery with NEAC takes place, is above the level agreed in the Norwegian management plan (ICES 2024). CC is considered more vulnerable than NEAC due to the risk of overfishing local populations, and fishing regulations therefore aim to move the fishery further off the coast and towards NEAC. The focus of most of the earlier genetic investigations in this region has been to understand the population structure of CC on the coast and use NEAC as a reference sample (Fevolden and Pogson 1997; Fevolden et al. 2012; Nordeide et al. 2011; Dahle, Quintela, et al. 2018; Johansen et al. 2020). In contrast, NEAC has never been subject to the same genetic scrutiny and thus far no study has looked at population genetic structure of cod in the Barents Sea with an extensive set of samples as we do here.

Hypotheses have been put forward that the NEAC may comprise more than one population. According to a study of historic cod roe landings, Sundby and Nakken (2008) found that in cold years NEAC migrated further south to spawn along the Norwegian coast while spawning further north in warm years. From this finding, they suggested that the NEAC from the east and west Barents Sea could reflect separate subpopulations with different spawning success depending on varying Barents Sea water temperatures among years.

The NEAC stock is monitored annually by the Norwegian Institute of Marine Research (IMR) in three surveys and stock identification is done by means of otolith typing for cod older than two years (Rollefsen 1933, 1934; Jakobsen 1987; Stransky et al. 2008). In addition to NEAC, a small fraction of CC, as identified by otoliths, are regularly observed in the Barents Sea winter surveys. However, these putative CC observations have so far been included in the numerical estimate of the NEAC stock (Godiksen et al. 2025). While all cod from the Barents Sea are assumed to spawn along the Norwegian coast, where they in some areas overlap in spawning sites with CC (Olsen et al. 2010; Johansen et al. 2020), oceanographic particle drift models indicate that only the NEAC eggs and larvae end up in the Barents Sea (Vikebø et al. 2005; Myksvoll et al. 2014). It is nevertheless unclear if some CC eggs drift into the Barents Sea. The two types of cod, CC and NEAC, are hereafter referred to as ‘ecotypes’, referencing their different life histories (Michalsen et al. 2014; Johansen et al. 2018; Strøm et al. 2023).

The distribution of cod ecotypes has been studied along the coast of Norway by both microsatellites (Skarstein et al. 2007; Westgaard and Fevolden 2007; Wennevik et al. 2008; Dahle, Quintela, et al. 2018) and Single Nucleotide Polymorphic markers (SNPs) (Nielsen et al. 2009; Fevolden et al. 2012; Hemmer‐Hansen et al. 2013; Kirubakaran et al. 2016; Berg et al. 2016, 2017; Johansen et al. 2018, 2020; Jorde et al. 2021). In the 1990s, the *Pan I* locus was found to display the highest differentiation between the NEAC and CC (Fevolden and Pogson 1997) and has been extensively used to separate the two ecotypes. These studies found that NEAC show high frequencies of the Pan I*BB genotype, with observed frequencies of the *B* allele in the range 0.78–1.00 (cf. Fevolden and Pogson 1997; Sarvas and Fevolden 2005; Westgaard and Fevolden 2007; Michalsen et al. 2014). In contrast, CC shows high frequencies of the alternate *Pan I* homozygote (*AA*) with the *A*‐allele frequency increasing from north to south from approximately 0.8 to near fixation (Dahle, Quintela, et al. 2018). The *Pan I* polymorphism, later localised to a large inversion on chromosome 1 (Kirubakaran et al. 2016), has therefore been used as an alternative or supplement to otolith typing to discriminate between NEAC and CC in management applications along the coast of Norway (Berg et al. 2005; Stransky et al. 2008; Dahle, Johansen, et al. 2018; Johansen et al. 2018). While *Pan I* can be used to assign pooled samples to CC and NEAC, the precision of this marker alone is not high enough for a fully correct individual assignment. Therefore, this marker combined with additional genetic markers has been used for individual assignment with a higher degree of accuracy (e.g., Michalsen et al. 2014; Johansen et al. 2018).

Some studies have compared otolith classification and genetic assignment of cod to ecotype (Berg et al. 2005; Stransky et al. 2008; Jorde et al. 2021; Wennevik et al. 2008; Johansen et al. 2018), but none of them has found a full match between the otolith and genetic assignment of individual cod to ecotype on the coast of Norway. For example, Jorde et al. (2021, their Table 3) found that a substantial fraction (18%) of cod that were caught off the Norwegian coast and carried CC type otoliths nevertheless genetically assigned to NEAC. This apparent mismatch between genetic and otolith methods could reflect biological or technical issues. Stransky et al. (2008) did the most systematic comparison between coastal cod and NEAC by comparing otolith and *Pan I* genotype and found that the variation in the otoliths likely reflected environmental influences.

In the present study we used a combination of otolith and genetic‐based classification of an extensive set of cod samples in the Barents Sea, Svalbard and the Northern Norwegian coast in order to address the following questions: (i) Are there more than one population of cod in the Barents Sea? and, specifically, (ii) are CC present in the Barents Sea and if so where and to what extent? For the first time we explore genetic profiles of NEAC stock from extensive sampling throughout the distribution area, representing the main spawning, nursery and feeding area for this stock.

## 2 Materials and Methods

### 2.1 Sampling

**FIGURE 1:**
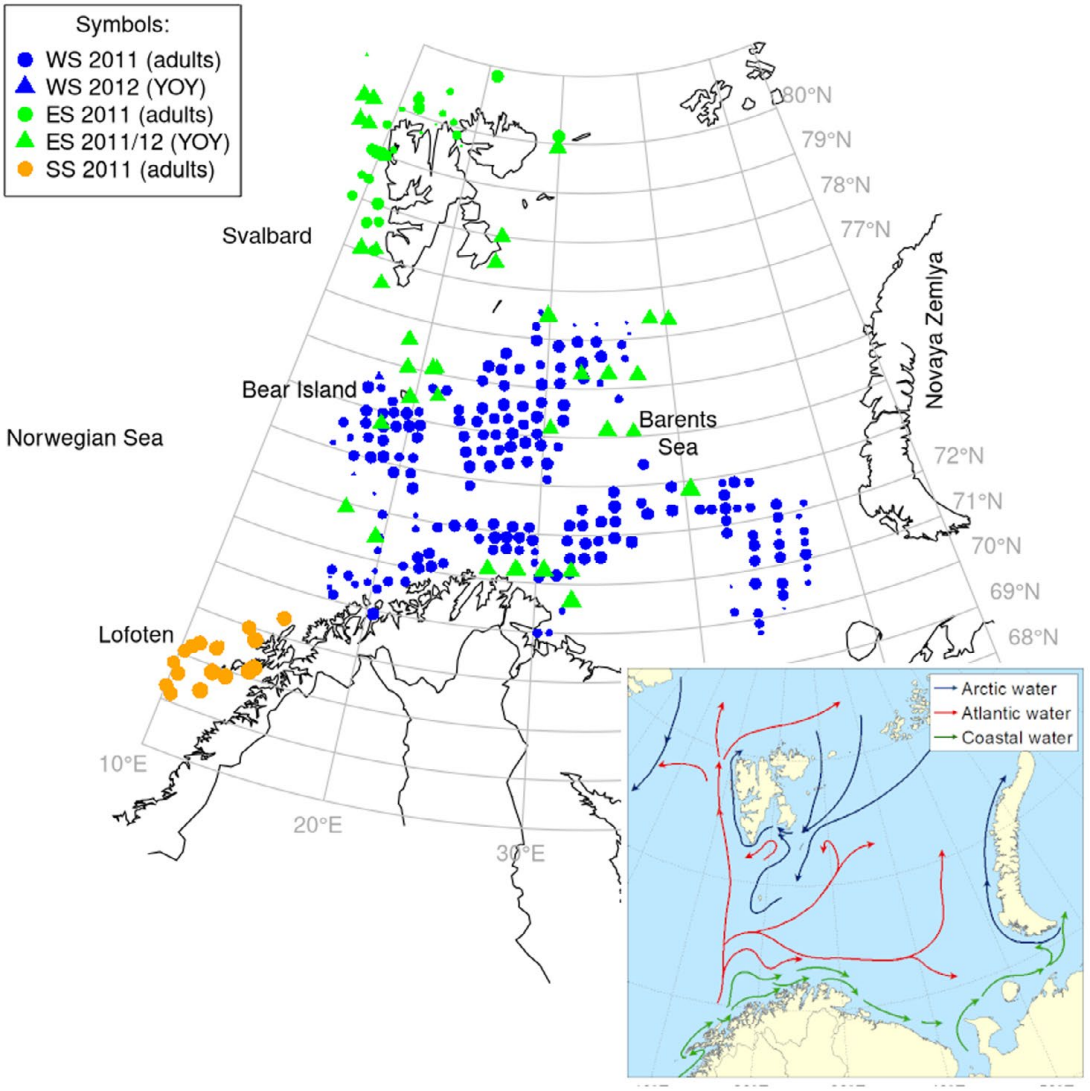
Sample locations in the Barents Sea and adjacent waters. Colours and symbols indicate survey type, year and age‐category (ad = adults, YOY = Young‐of‐the‐Year: Cf. Table 1), respectively. Each symbol is one station and the symbol size is scaled to log_e_ number of individuals. WS: Winter survey, ES: Ecosystem survey, SS: Spawning survey. See Table 1 for details on samples. Inset: Ocean currents (arrows).

During 2011 and 2012, a total of 3900 adult and juvenile cod were collected during three routine surveys in Northern Norway and the Barents Sea (Table 1). The surveys are conducted every year to monitor the Northeast Arctic cod (NEAC) stock by the Institute of Marine Research (IMR) in Norway and include the following: The Winter survey (WS) and the Ecosystem survey (ES) that are trawl‐acoustic surveys taking place in the Barents Sea in February–March and August–October, respectively. The Spawning survey (SS) taking place in Troms‐Lofoten in March–April and monitoring the migratory spawning stock primarily using acoustic methods. While the two early surveys (WS, SS) collect biological samples (age, individual weight, maturity stage, sex, etc.) of cod one year and older, the ES survey also collects information about the age zero group (Table 1, Figure 1).

**TABLE 1:** Atlantic cod samples collected from four assessment surveys (IMR‐surveys) of adult and YOY (young‐of‐the‐year or 0‐group) cod over two years.

Area	Survey/season	Year	IMR survey	No stations	Age‐class (range years)	Analysed			
						Otoliths	Pan I + 3 msats^a^	6 msats^b^	36 SNPs^c^
Troms‐Lofoten	Spring/spawning	2011	SS2011	19	adult (2‐14)	863	863	384	169
Barents Sea	Winter/feeding	2011	WS2011	198	adult (1‐13)	1662	1741	1166	161
		2012	WS2012	3	YOY	0	102	102	0
	Ecosystem/autumn	2011	ES2011	56	adult (1‐9)	203	253	253	4
					YOY	0	487	487	236
	Ecosystem/autumn	2012	ES2012	19	YOY	0	454	355	0
Sum				295		2728	3900	2747	570

*Note:* The number of cod typed for otoliths and genotyped (for *Pan I* and 3 microsatellites, for 6 additional microsatellites and for 36 SNPs) are given, along with range of age‐classes and number sample locations (stations) covered in each survey.

^a^ *GMO3*, *GMO34* and *GMO132*.

^b^ *GMO2*, *GMO8*, *GMO19*, *GMO3*5, *TCH11* and *TCH13*.

^c^ See list of SNPs in Table S2. See text for details on the sampling design and Figure 1.

In the above‐mentioned surveys, biological samples of cod are taken in a stratified manner to get representative age‐length keys. Stratification is based on fish length, as follows: For the WS, one cod is collected for each 5 cm length class from each trawl station (Godiksen et al. 2025). For the SS, 10 cod for every 5 cm from each station are collected. In the present study, we took genetic samples from these same cod to get unique and management‐relevant insight into the presence of the two ecotypes in the Barents Sea. This resulted in 2–20 sampled fish from 19 to 300 stations depending on the survey (cf. Figure 1). We were also interested in the genetic profiles of young‐of‐the‐year (YOY) and therefore supplemented the standard sampling with random samples from the ES (autumn 2011 and 2012). From each fish, gill tissue was collected for DNA analysis and otoliths for age and ecotype classification. The present genetic analyses thus cover all cod from the statistical population collected in the mentioned surveys.

### 2.2 Otolith Classification

Using otoliths, all cod were classified to ecotype and aged onboard the survey vessels by a trained expert. The ecotype classification of otoliths is based on the principle that the two inner translucent annual zones differ between NEAC and CC types, as described in Rollefsen (1933) and modified in Berg et al. (2005). Otolith types 1 and 2 are characterised as ‘certain’ and ‘uncertain’ CC respectively; 4 and 5 denote ‘uncertain’ and ‘certain’ NEAC. Otolith type 3 denotes Svalbard cod but is here ignored as only two individuals carried this type and use of this type has been discontinued. Otolith typing of cod cannot be performed on individuals younger than 2 years of age and so it was not performed on YOY fish, that is, on most of ES2011 and all of ES2012 cod (cf. Table 1).

A subset of 297 otoliths was selected for reanalysis in the laboratory to check the reading consistency of the on‐board otolith typing and to check some apparent mismatches between typing based on otolith and genetics observed in preliminary analyses. The age of these cod spanned from 2 to 10 years and individuals were chosen in approximately equal numbers from each of the WS (*n* = 127) and SS surveys (*n* = 170). Reanalysis was carried out by retyping otoliths in the lab by two trained experts at IMR using a randomised, blind design. Otoliths were put in anonymous envelopes, given a randomised number without any information on sampling site, type at survey or other biological characteristics, and scored independently by the two experts. We compared the score between experts and counted the number of matches when they agreed or disagreed on otolith types.

### 2.3 Genetic Analysis

A total of 3900 cod from the described surveys were analysed for *Pan I* and three microsatellites, *GMO132, GMO34* and *GMO3* (Table 1). Most samples (2747 cod) were also genotyped with a further six microsatellites, but only 570 cod were analysed with 36 SNPs to test the assignment rate to ecotype (Table 1—for explanation see below). The study focussed on microsatellite markers that have previously been found to display highly divergent allele frequencies between NEAC and CC (Westgaard and Fevolden 2007; Wennevik et al. 2008; Michalsen et al. 2014). Positions in the genome (Table S1) for all markers were inferred from results of running nucleotide‐nucleotide BLAST (Altschul et al. 1990) on the primer sequences (Brooker et al. 1994; Miller et al. 2000; Pogson 2001; Johansen et al. 2018) against the latest (gadMor3) genome assembly (Star et al. 2011; Tørresen et al. 2017; Anon 2019: NCBI accession ID GCF_902167405.1). The Atlantic cod genome possesses four large, polymorphic chromosome inversions on chromosomes 1, 2, 7 and 12 (Sodeland et al. 2016; Kirubakaran et al. 2016) and these inversion regions represent the most strongly divergent parts of the Atlantic cod genome between CC and NEAC (Berg et al. 2016; Johansen et al. 2020). Hence, genetic markers in this region are particularly useful for separating NEAC from CC, and all four inversion regions are covered by the panel of 36 SNPs (Table S1). Historically, the *Pan I* locus has been widely applied for this purpose (Fevolden et al. 2012, Johansen et al. 2018; Dahle, Quintela, et al. 2018) and along with *GMO34* (both located within distal parts of the inversion on chromosome 1) and *GMO13*2 (chromosome 7) constitute the major genetic tools in the present study.

Genetic differences among surveys, sample years and age classes were tested for by heterogeneity Chi‐square tests of allele frequencies (*qhisq.test* in R, without continuity correction: R Core Team 2022). These and further statistical tests employing microsatellites were carried out after pooling minor alleles to minimise statistical type I errors caused by low expected values for low‐frequent alleles. Spatial heterogeneity in allele frequencies was tested for deviation from the means over the study area in the Barents Sea (excluding near‐coast sites). To avoid very small sample sizes, sample stations were binned into cells of 1 degree latitude by 3 degrees longitude, corresponding to about 110 by 90 km at the centre of the present study area (at 74° N, 30° E) and cells with less than 10 individuals were omitted.

The set of 36 SNPs, scored in a subset of 570 individuals, was used to partition individual cod into putative CC and NEAC clusters with the STRUCTURE software (Pritchard et al. 2000). These fish included the 297 cod that were subject to the otolith re‐screening described above, 240 YOY from ES2011 + ES2012. Some 33 adult fish from survey ES2011 were also included in the SNP genotyping for practical reasons as they were found on the same DNA plate. This panel of SNP markers was originally developed to differentiate between NEAC and CC, and for characterising variation among CC on the coast of Norway (Johansen et al. 2018). Of the 36 SNPs, 11 reside within the large inversion on chromosome 1, and 4 within each of the inversions at chromosome 2, 7 and 12 (cf. Table S1). We chose the ADMIX model with correlated allele frequencies as the biologically most realistic scenario and explored both K = 2 and K = 3 populations, using the running parameters of 300,000 burn in and 1,000,000 MCMC. The runs were repeated 10 times and summarised with the CLUMPP v. 1.1.2 software (Jakobsson and Rosenberg 2007). Clustering of individual cod based on the 36 SNPs was also explored with the discriminant analysis of principal components (DAPC) approach, using the *find.clusters* and *dapc* functions (Jombart et al. 2010) in the *adegenet* package (Jombart 2008) in R. In this analysis we retained all (36) PCs and exploited various numbers of clusters (K = 2 to 5).

### 2.4 Comparison of Otolith Versus Genetic Assignment to Ecotype

We compared classifications of individual cod to population‐of‐origin (CC or NEAC) from otolith typing to genetic assignments based on STRUCTURE K = 2 analyses for the 297 individuals that were screened with SNPs and also included in the otolith‐retyping experiment. Individual growth of cod carrying CC and NEAC otoliths was compared using the von Bertalanffy growth function:

$$\text{Length}\sim {\text{Linf}}^{*}\left(1-\exp\left(-k^{*}\mathrm{Age}-\text{t0}\right)\right),$$

where Linf, k and t0 are growth parameters, estimated by fitting observed individual Length and Age with non‐linear least squares using the *nls* function in R.

## 3 Results

### 3.1 Otolith Types

**FIGURE 2:**
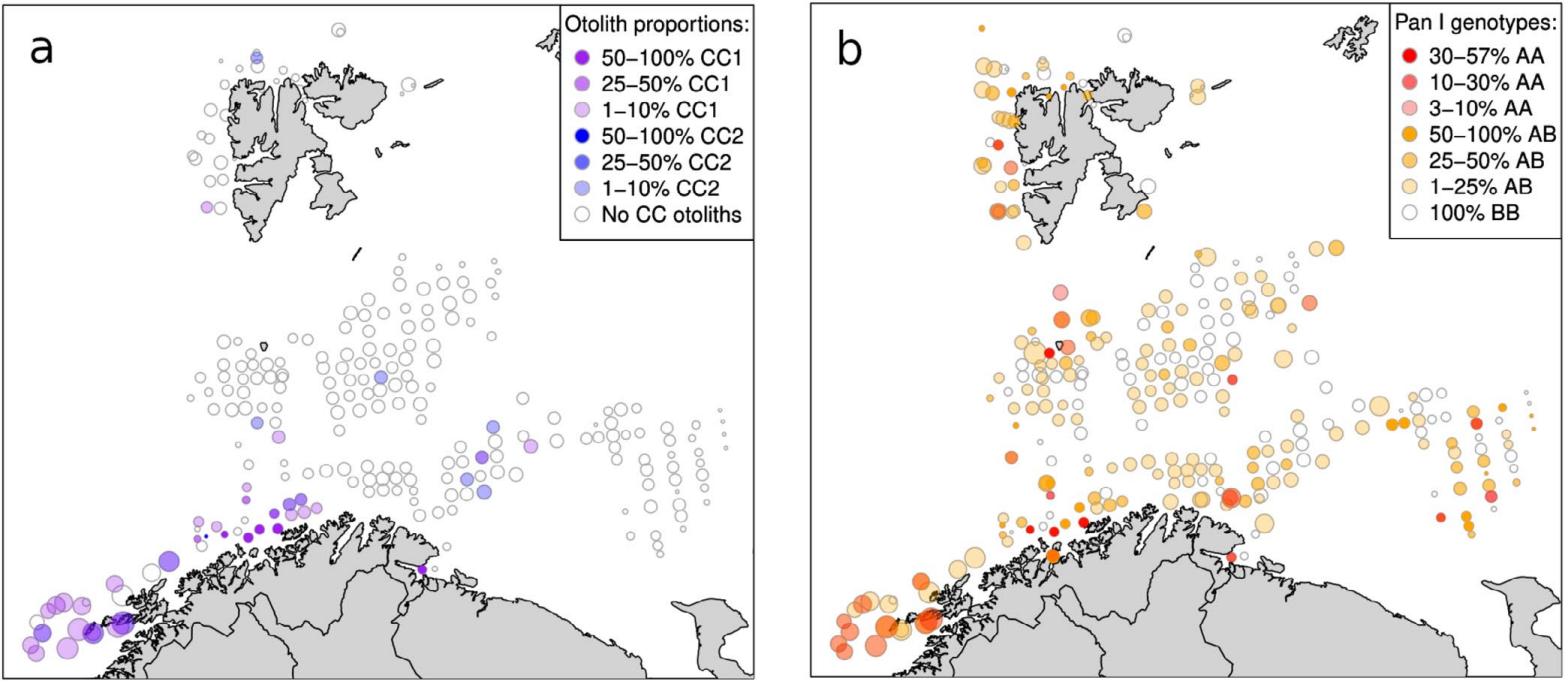
(a) Proportions of individuals that were otolith‐typed as coastal cod (CC type 1 [*n* = 69]; purple; uncertain CC type 2 [*n* = 20]; blue). (b) Proportions of individuals carrying the *Pan I*‐A allele (homozygote *AA* [*n* = 58]: Red; heterozygote *AB* [*n* = 580]: Orange). Sizes of dots are drawn in proportion to log_e_ sample size (sample sizes ranged from 1 to 96). Young fish (age < 2) were not otolith‐typed and therefore not included in the a panel.

Based on otolith‐reading, 2728 cod aged 2 year or older were type‐classified onboard the survey vessels (Table 1), yielding 2546 fish judged as ‘certain NEAC’ (otolith type 5) and 69 as ‘certain CC’ (type 1), whereas 111 were uncertain (20 type 2 and 91 type 4). This represented an average of 2.6% CC in total, with a similar proportion in the Winter survey (WS2011: 2.1%) but somewhat higher in the Spawning survey (SS2011: 3.7%). The spatial distribution of otolith types throughout the study area was heterogenous, with CC reaching high proportions at some near‐coast locations whereas only a few CC otoliths were found offshore in the Barents Sea and around Svalbard (Figure 2a). Fish with CC otoliths were on average younger than NEAC in the same survey (SS2011: mean age 5.47 vs. 7.38 years; WS2011: 4.06 vs. 4.47 years, for CC and NEAC, respectively), although only the former was statistically significant (the *t.test* function in R gave *p* = 3.17*10^−6^ and *p* = 0.200, respectively). Fish carrying CC otoliths also displayed faster growth during young age (< 5 year, i.e., before maturation) than those with NEAC otoliths (Figure S1).

For the subset of 297 otoliths that were re‐typed in the laboratory by two experts in a blind test, 100% agreement was observed in otoliths that both experts had typed as ‘certain’, that is, either CC (type 1: 48 individuals) or NEAC (type 5: 171) (Table S2). Differences arose among the 78 individuals that either or both experts had labelled as ‘uncertain’ (type 2 or 4), and these otoliths were subject to a third reading in order to arrive at a census type. Comparing the census otolith types at the lab with the original types done onboard the survey ships revealed 11 (4.9%) discrepancies out of 225 ‘certain’ types (Table 2). Taking the census type as ‘correct’, this implies an error on on‐board otolith typing of nearly 5%.

**TABLE 2:** Assessment of survey otolith typing reliability based on re‐typing of 297 otoliths in a blind laboratory experiment.

On‐board otolith type	Lab census otolith type			
	CC		NEAC	
	Certain (1)	Uncertain (2)	Certain (5)	Uncertain (4)
Certain CC (type 1)	56	7	5	0
Uncertain CC (type 2)	5	4	5	5
Certain NEAC (type 5)	6	4	158	5
Uncertain NEAC (type 4)	5	6	20	6

*Note:* Lab type refers to census type arrived at by the two lab.

### 3.2 Genotyping

In total, 3900 adult and YOY cod were genotyped for *Pan I* and the three microsatellites *GMO3*, *GMO34* and *GMO132*. *Pan I* is biallelic whereas the three microsatellites segregated in the present material for 15, 15 and 33 alleles, respectively. The major allele at each locus reached a high frequency in the total sample: Pan I*B allele = 0.909; GMO3*191 = 0.915, GMO34*98 = 0.931 and GMO132*116 = 0.740. For all four loci there was a deficiency of heterozygotes relative to expectations under Hardy–Weinberg genotype equilibrum (HWE), but statistically significant only for *Pan I* (*F*_IS_ = 0.083; X^2^ goodness‐of‐fit test: *p* < 0.0001). Splitting the samples into surveys revealed that the departure from HWE at *Pan I* was largely confined to the SS survey (*F*_IS_ = 0.187; *p* < 0.0001), likely reflecting population mixture (Wahlund effect) within the SS sample. In contrast, there was weak, if any, deviation within the other two surveys (pooling years: ES2011 + 2012: *F*_IS_ = 0.027; *p* = 0.34, WS2011 + 2012: *F*_IS_ = 0.05; *p* = 0.37). Removing individuals with CC otoliths reduced the deviation from HWE in the SS survey to a non‐significant level (*n* = 817; *p* = 0.058), strengthening the evidence for mixture of CC and NEAC cod in catches from this survey.

**FIGURE 3:**
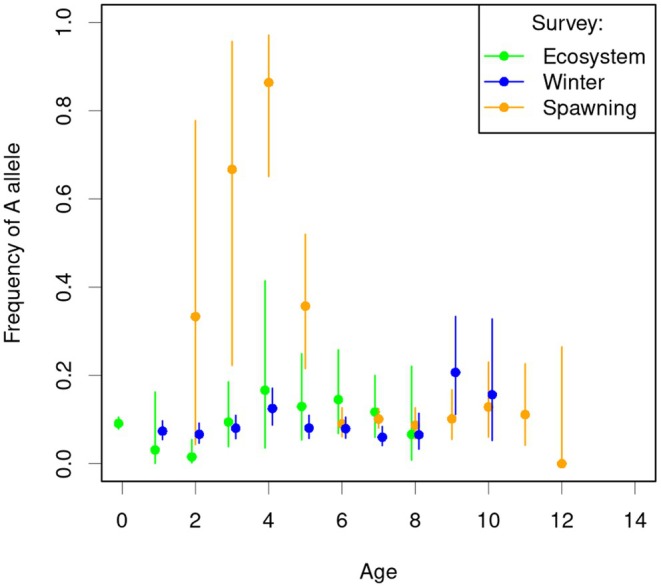
Pan I*A‐allele frequency among age‐classes (older age classes pooled due to small sizes) for the three surveys. Vertical bars represent exact 95% binomial confidence intervals, calculated with the *binom. test* function in R.

Temporal sampling in 2011 and 2012 from the Barents Sea did not reveal any significant allele frequency difference between sample years at any of the four loci in either the adults collected in winter (WS) or the YOYs from the autumn (ES) surveys. However, comparing *Pan I* allele frequencies among age classes revealed statistically significant differences among age classes within each survey in heterogeneity Chi‐square tests: Spawning survey (13 age classes; pooling the 3 oldest classes because of small numbers): *X* ^2^ = 171.78, df = 10, *P* < < 0.0001; ES survey (10 age classes; pooling the 2 oldest classes): *X* ^2^ = 15.92, df = 8, *p* = 0.044; Winter survey (13 age classes; pooling the 4 oldest classes): *X* ^2^ = 27.43, df = 9, *p* = 0.0012. The difference was especially pronounced in the Spawning survey with a high frequency of the minor (*A*) allele in younger (immature) age classes (Figure 3). From otolith typing, these young individuals were inferred as most likely CC, as 16 out of 17 carried a CC otolith type (type 1 or 2) and one carried an (uncertain) NEAC type 4.

Spatial analyses of *Pan I* genotypes revealed uneven distribution of genotypes throughout the study area (Figure 2b). Pan I *A‐bearing genotypes (i.e., homozygote *AA* and heterozygote *AB*) occurred in moderate to high frequencies along the Norwegian coast, around Bear Island, Svalbard and in the far eastern Barents Sea. Samples from most of the central Barents Sea, in contrast, consisted almost exclusively of *BB* homozygotes with heterozygotes occurring in less than 10% (117 out of 1131 individuals) in the central region between 72° –77° N and 20° s–40° E, while AA homozygotes were nearly absent (just 2 individuals). Tests for allele frequency deviations in pooled samples (cells of 1 degree latitude by 3° longitude) yielded highly significant departures from the mean frequency: in near‐coast samples the deviations were in the direction of higher‐than‐average frequency of *A*, whereas elsewhere departures occurred in either direction (Figure S2). This test, as well as subsequent ones for the microsatellites (below), refers to goodness‐of‐fit for frequency of the major allele to the average frequency over the Barents Sea, that is, in the pooled total material excluding near‐coast samples (*n* = 1107) defined as cells that overlapped the coastline (red line in the Figure S2: using coordinates from https://lovdata.no/dokument/SF/forskrift/2002‐06‐14‐625).

As for *Pan I* (above), tests of major allele frequency deviations were carried out for microsatellites separately in geographical sites defined by pooling samples into bins of 3 degrees latitude and 1 degree longitude, and multiple tests were combined over samples (bins) by summing Chi‐square values. Results show highly significant overall allele frequency deviations for *GMO34*, with near‐significant results for *GMO132* (summed over bins: *p* = 0.067) and *TCH11* (*p* = 0.087). Deviations occurred most frequently in near‐coastal sites, with significantly lower frequency of the major allele at *GMO34* and *GMO132*, mirroring the *Pan I* results above. Six additional microsatellites, scored at the majority of samples (2747 of 3900 sampled cod: Table 1), were also included in tests for spatial genetic structure and revealed significant allele frequency deviations (in either direction) in several offshore sites but with no obvious spatial pattern (cf. Figure S2). Out of 365 tests involving these 6 loci, 13 tests (3.6%) were significant at the nominal 5% level and 6 of them also at the 1% level of significance.

### 3.3 Individual Classification Using SNPs

**FIGURE 4:**
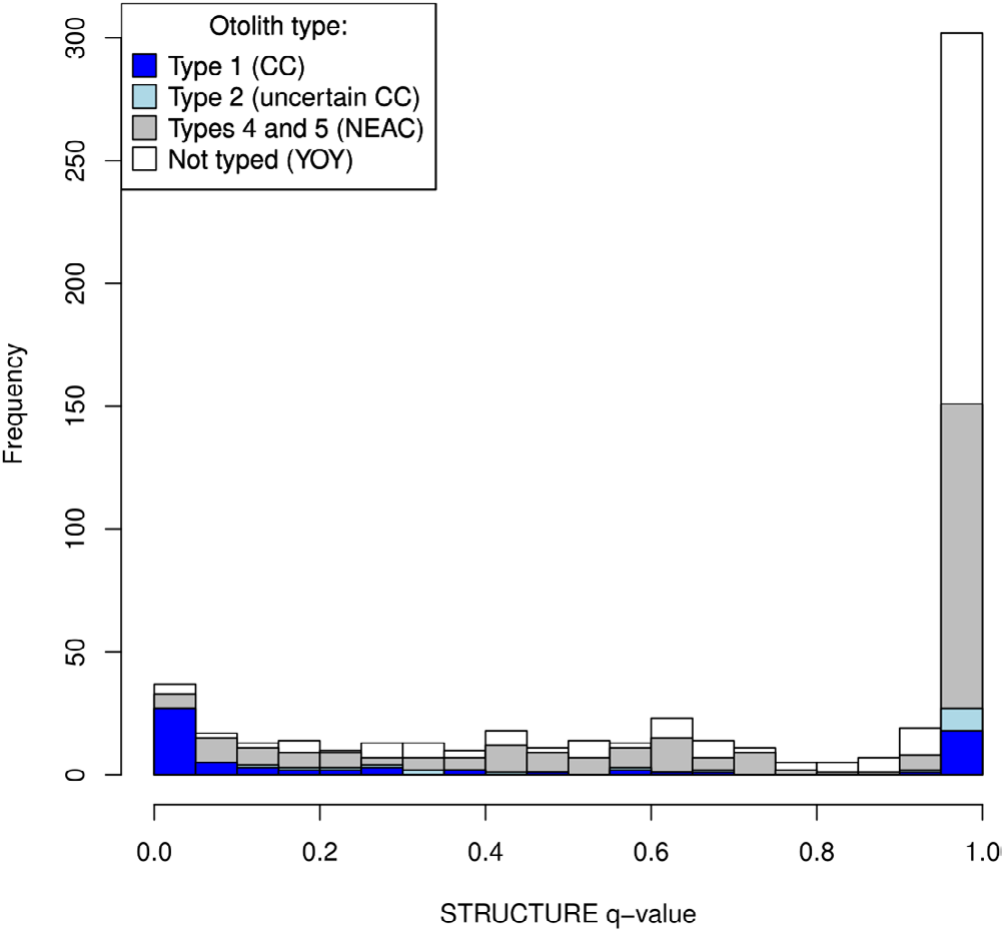
Distribution of STRUCTURE K = 2 *q* values among 570 fish genotyped for 36 SNPs (admix model with correlated allele frequencies). The highest peak (> 0.8) were dominated by NEAC otolith types (type 5 or 4: Grey) and assumed NEAC whereas the low peak (< 0.2) consisted mostly of individuals carrying CC otolith type 1 (blue) and inferred as CC. (YOY) individuals were not otolith typed (white).

A panel of 36 SNPs were screened in a subset of 570 individuals (cf. Table 1) to characterise likely population‐of‐origin with STRUCTURE. The results indicated K = 2 populations as most likely, with one large and one small peak in *q* values near 1 and 0, respectively (Figure 4). Cross‐checking against otolith types (*n* = 297) identified the smaller peak as CC, as most individuals in this peak had otolith type 1 (i.e., certain CC), and the major peak as NEAC since the majority of otolith‐typed individuals in this STRUCTURE class carried NEAC (type 5) otoliths. However, a number of individuals with type 1 and 2 otoliths (19 and 10, respectively) were also included in the NEAC class by STRUCTURE. There was also a relatively large number of individuals (152) with intermediate *q* values (between 0.2 and 0.8) and therefore left unassigned to population‐of‐origin in the STRUCTURE analysis.

Using the same data (*n* = 570, 36 SNPs) in DAPC we found no obvious pattern in the Kstat profile to aid in finding the optimal number of clusters and chose K = 2 for comparison with STRUCTURE results. DAPC thus identified two asymmetrically sized clusters, with 524 and 46 individuals, respectively. Comparison with STRUCTURE (Figure S3) revealed that all members of the DAPC minor cluster fell within the CC group whereas the DAPC major cluster included some CC as well as all individuals classified as NEAC and uncertain by STRUCTURE. Neither approach was able to separate all individuals into distinct, non‐overlapping clusters.

### 3.4 Comparison of Otolith and Genetic Assignment to Ecotypes

Assignment of individuals to NEAC and CC types by otoliths on one hand and genetic assignment by STRUCTURE on the other, was done for 297 individuals for which both otolith and SNP data were available. Limiting our attention to individuals that were considered ‘certain’ with respect to both otolith types (i.e., types 1 and 5) and genetic assignment, there were highly discordant numbers between the otolith and genetic assignment methods, as only 38 of 64 individuals genetically assigned to CC had a matching CC (type 1) otolith (Table 3) whereas 22 individuals instead had NEAC (type 5) otoliths (contingency Chi‐square test, X^2^ = 45.51, df = 1, P < < 0.0001). These 22 individuals, genetically CC, occurred throughout the study area including 7 cod far offshore in the Barents Sea (cf. Figure S4a). Conversely, 18 individuals carried a CC otolith type despite being classified genetically to the NEAC cluster (Figure S4b). These numbers refers to census otolith types from the lab. Compared with on‐board otolith typing, there were only slight differences in terms of numbers but on‐board typing yielded fewer CC types out in the Barents Sea than did lab‐typing (cf. compare Figure S4b,d). This observation could indicate a tendency for on‐board otolith typing being influenced by information about the area where the fish is collected, which was intentionally hidden in the lab re‐typing experiment.

**TABLE 3:** Comparison of genetic assignments and otolith type among 297 experimental individuals that were SNP‐genotyped.

Genetic assignment	Lab census otolith type			
	CC		NEAC	
	Certain (1)	Uncertain (2)	Certain (5)	Uncertain (4)
CC	38	1	22	3
NEAC	18	8	106	8
Uncertain	16	12	59	5

*Note:* Genetic assignments were performed in STRUCTURE using 36 SNPs with assignment criteria (cut‐off *q* values) of 0.2/0.8. Lab type refers to census type arrived at by the two lab technicians. For geographic distribution of genetic/otolith‐type mismatches see Figure S4a,b.

## 4 Discussion

This is the first study to explore genetic structure in Atlantic cod sampled in both the main nursery and feeding areas for the NEAC stock in the Barents Sea, and the NEAC spawning run to the Norwegian coast. Our main finding is that there are members from more than one population of cod present in the Barents Sea and around Svalbard, with CC mixed in the numerically dominant NEAC throughout the Barents Sea but in varying proportions (Figure 2a,b). Moreover, otoliths are not a good indicator of genetic ancestry of cod but seem largely influenced by the environment. Below, we discuss the rationale for and implications of these observations.

Evidence for more than one population of cod follows from our finding of genetic heterogeneity within the Barents Sea as well as along the coast. Genetic heterogeneity was statistically highly significant only for *Pan I* and *GMO34*, that is, loci situated on the large inversion on chromosome 1 which is the part of the genome that most clearly separates CC from NEAC (Berg et al. 2016; Johansen et al. 2020). The results of the STRUCTURE analyses indicated that the observed genetic heterogeneity most likely can be explained by the presence of both CC and NEAC in the samples. Using *Pan I* genotypes as a ‘trace’ of origin, its heterogenous distribution indicates presence of CC along the Norwegian coast as expected (Jorde et al. 2021; Dahle, Quintela, et al. 2018; Breistein et al. 2022), but also into the Barents Sea towards Bear Island and Svalbard in the west and also in the far eastern Barents Sea (cf. Figure 2b), whereas the central Barents Sea is largely void of CC. We speculate that this heterogenous distribution is caused by transportation by ocean currents (cf. Figure 1 insert) of pelagic CC eggs and larva from downstream spawning at the Norwegian coast. Because our sampling only covers 2 years, the temporal stability of this novel observation is unclear and should be followed up in new studies.

Individuals that failed to be classified genetically to population of origin, that is, by having intermediate STRUCTURE *q* values (cf. Figure 4) may include CC × NEAC hybrids and backcrosses. DAPC also failed to clearly separate individuals into distinct classes and the minor cluster (presumed CC) had a wide spread in coordinate values (cf. Figure S3). Future studies using whole genome sequencing should search for independent (i.e., outside the chromosome 1 inversion region) informative genetic markers to resolve ancestry relationships among cod.

A second important finding of the current study was the observation that otolith type, routinely used to differentiate between CC and NEAC in surveys and in biological samples from the Norwegian fisheries (ICES 2024), did not always match the genetic profile of the individual. Instead, otolith type tended to follow geography, with few cod displaying CC‐type otoliths being found more than 50 km from land (cf. Figure 2a). A number of these offshore CC‐otolith bearing individuals did indeed have a matching CC genotype profile (*Pan I* genotype) and were thus most likely true coastal cod despite being captured tens of km from the coast. In several cases, however, there was a mismatch between genetic (SNP‐based) and otolith type classifications, with some individuals being classified genetically to CC despite having a NEAC‐type otolith profile and others being classified genetically to NEAC yet carrying CC‐type otoliths (Table 3; Figure S4). Together, this apparent methodological mismatch in type, as well as the contrasting geographic distribution of otolith and genotypes, is consistent with the notion that otolith types reflect differences in the environment of the nursery area rather than population of origin (Stransky et al. 2008; Jorde et al. 2021; Spotowitz et al. 2022). This finding raises questions on the validity of otoliths as a means for classifying individual cod to population of origin, especially in a stock management context.

A third finding of this study was that both genetic assignment to population of origin and otolith‐typing itself are associated with uncertainties and potential errors. Blind retyping of otoliths yielded different classification for 11.4% of individuals that were scored as certain otolith types on‐board (on board 68 were scored as certain CC vs. 56 in the lab and 173 as certain NEAC vs. 158 in the lab: Table 2), although much of this reclassification refers to a shift from certain to uncertain types (2 or 4). Only 11 individuals (4.9%) received a directly conflicting type on‐board as compared to the lab census type: six NEAC were reclassified as CC and five CC reclassified as NEAC. Hence, otolith typing is unlikely to explain the difference in ecotyping as compared to genetics and a putative < 5% error rate should probably be considered acceptable.

The relatively large fraction of individuals (156 out of 570 or 27.4%) left unassigned by genetic methods (STRUCTURE) could indicate insufficient statistical power of resolution, problems with physical linkage within inversions, and/or hybridisation between the CC and the NEAC (Spotowitz et al. 2022). The applied panel of SNP markers, although designed to distinguish between NEAC and CC, is not able to unravel hybridisation patterns to the same extent as full‐genome sequencing (Breistein et al. 2022). Nevertheless, potential errors in genetic ecotype classification cannot explain the large, and spatially varying, presence of CC‐type *Pan I* and *GMO34* genotypes through the study area (cf. Figure S2) as these markers were not used in the STRUCTURE classification. The discrepancy in genetic and otolith type‐classification is therefore a real phenomenon, most likely due to the environmental impression on otoliths.

The first sampling year of this study in 2011 turned out, in retrospective, to be a special year for the NEAC stock. In that year, an increasing fraction of NEAC was detected spawning as far south as 62° N (Dahle, Johansen, et al. 2018; Johansen et al. 2018), while this fraction decreased again from 2015 (Johansen et al. 2018). These changes are partly correlated with changes in abundance of the NEAC stock, which has suffered poor recruitment for the last 10–15 years (Ma et al. 2024; ICES 2024; Johannesen et al. 2026). Then, an important question is the possible role of hybridization between the two stocks, due to increased overlap in spawning areas, and whether hybrids could have a significant impact on cod population dynamics in the Barents Sea. In light of these changes and our results, we propose further genetic studies on changes in overlap with CC in spawning areas and the possible role of hybridization between the two ecotypes in affecting the respective population dynamics. In that sense, further tagging studies combined with genetic tools would be an important tool (Michalsen et al. 2014; Strøm et al. 2023; Svåsand et al. 2025).

### 4.1 Monitoring and Management Implications

Optimal exploitation of fishery resources requires that biological populations with distinct demographic rates are identified and separately assessed. Our finding of genetic heterogeneity among cod within the Barents Sea, also when leaving coast‐near areas out of the analyses, implies that the present management and harvesting regime that treats all cod in the Barents Sea as a single (NEAC) population may fail to adequately protect the CC resources. However, several questions remain to be answered, such as whether the cod of genetic CC origin identified offshore have adapted to the life of a NEAC, and whether the offspring of such cod will be a part of the NEAC or Norwegian CC populations. If cod of genetic CC origin have advected into the Barents Sea as juveniles and are behaving as the migrating NEAC ecotype, the current management regime that considers these cod as part of NEAC may be appropriate. We propose to initiate regular genetic monitoring of the stock to get a better overview of the fate, frequency and temporal stability of the genetic CC and potential genetic CC‐NEAC hybrids in the Barents Sea.

Finally, we demonstrate that otoliths, which seem mainly to reflect oceanic versus coastal habitat, have shortcomings as a marker for population of origin and should ideally be supplemented with genetic sampling for ecotype classification. The present genetic markers yielded insufficient precision in assigning individual fish with high precision, and work should be initiated to improve on the set of markers to better separate ecotypes and detect potential hybrids.

## Funding

This work was supported by IMR and funded the project.

## Conflicts of Interest

The authors declare no conflicts of interest.

## Supporting information


**Figure S1:** Age and body length (dots) and estimates of von Bertalanffy growth function (lines) in cod classified as ‘CC’ or ‘NEAC’ based on otolith types (readings from surveys: NEAC (type 5), *n* = 2546; CC (type 1), *n* = 69).
**Figure S2:** Spatial tests for allele frequency deviations in 9 microsatellites and Pan I from the Barent Sea average frequency (based on 2793 cod caught off‐shore from the red coastline). Samples are binned into cells of 1 degree latitude and 3 degrees longitude. For cells that do not overlap the red coastline, average allele frequencies (pooling minor alleles at microsatellite loci) were calculated and used in tests for allele frequency deviations. For each cell, observed allele frequencies were tested for goodness‐of‐fit to these averages using a Chi‐square test with df = 1. The test result for each cell is coloured according to significance level (red for α < 0.05, brown for < 0.01) and are not adjusted for multiple tests (cells). The direction of major allele frequency deviations from the mean are indicated by a sign (+/−) whether statistically significant or not. Multiciply of tests (cells) is implicitly accounted for in a global test, summing Chi‐square values over all cells, with details given in the title for each panel (locus).
**Figure S3:** Classification of 570 cod (open circles) based on 36 SNPs into K = 2 groups, with STRUCTURE (horizontal axis) and DAPC (vertical axis). Marginal histograms represent the disribution of individual statistics (STRUCTURE q values or DAPC coordinates) with bars coloured according to inferred biological populations (NEAC: red; CC: blue). In STRUCTURE, individuals with intermediate q values (between 0.2 and 0.8) were left unassigned (grey bars).
**Figure S4:** Position of cod with mismatching ecotype classifications based on otolith versus genetics (STRUCTURE K = 2 classes, using 36 SNPs). Left panels (a + c): Otolith type NEAC (type 5) but genetically CC. Right panels (b + d): Otolith type CC (type 1) but genetically NEAC. Top panels (a + b): on‐board otolith typing. Bottom panels (c + d): lab census otolith typing. Coloured circles: position of cod with otolith‐genetics type conflicts (darker colours indicates > 1 individual). Open circles: positions with no otolith‐genetics type conflicts.
**Table S1:** List of genetic markers (SNPs and microsatellites) used in the present study, with chromosome number (Chr) and nucleotide position (start position of primer) in the Atlantic cod genome (version gadMor3). (For primer sequences see Johansen et al. 2018).
**Table S2:** Comparison of otolith classifications between two experienced lab‐technicians (Reader #1 and #2) in blind experiment. The same set of 297 otoliths were classified as CC or NEAC with high (types 1 or 5, resp.) or low (types 2 or 4) confidence. Differences in classifications between reader #1 and #2 occurred only for otoliths (78 in total) that were judged as uncertain by at least one.

## Data Availability

Data on which this study is based can be downloaded at: https://doi.org/10.83172/4x9f‐d385.
