# Supplementary material for: Coexisting Atlantic Cod Ecotypes in the Barents Sea: An Issue for Managing Fisheries
Source: Evol Appl. 2026 May 10;19(5):e70250. doi: 10.1111/eva.70250 (PMC13158372; doi:10.1111/eva.70250)
Supplement: Supplementary file 1 — Figure S1: Age and body length (dots) and estimates of von Bertalanffy growth function (lines) in cod classified as ‘CC’ or ‘NEAC’ based on otolith types (readings from surveys: NEAC (type 5), n = 2546; CC (type 1), n = 69). Figure S2: Spatial tests for allele frequency deviations in 9 microsatellites and Pan I from the Barent Sea average frequency (based on 2793 cod caught off‐shore from the red coastline). Samples are binned into cells of 1 degree latitude and 3 degrees longitude. For cells that do not overlap the red coastline, average allele frequencies (pooling minor alleles at microsatellite loci) were calculated and used in tests for allele frequency deviations. For each cell, observed allele frequencies were tested for goodness‐of‐fit to these averages using a Chi‐square test with df = 1. The test result for each cell is coloured according to significance level (red for α < 0.05, brown for < 0.01) and are not adjusted for multiple tests (cells). The direction of major allele frequency deviations from the mean are indicated by a sign (+/−) whether statistically significant or not. Multiciply of tests (cells) is implicitly accounted for in a global test, summing Chi‐square values over all cells, with details given in the title for each panel (locus). Figure S3: Classification of 570 cod (open circles) based on 36 SNPs into K = 2 groups, with STRUCTURE (horizontal axis) and DAPC (vertical axis). Marginal histograms represent the disribution of individual statistics (STRUCTURE q values or DAPC coordinates) with bars coloured according to inferred biological populations (NEAC: red; CC: blue). In STRUCTURE, individuals with intermediate q values (between 0.2 and 0.8) were left unassigned (grey bars). Figure S4: Position of cod with mismatching ecotype classifications based on otolith versus genetics (STRUCTURE K = 2 classes, using 36 SNPs). Left panels (a + c): Otolith type NEAC (type 5) but genetically CC. Right panels (b + d): Otolith type CC (type 1) but geneti [file EVA-19-e70250-s001.docx]

**Electronic supplement**

**Suppl. Table S1:** List of genetic markers (SNPs and microsatellites) used in the present study, with chromosome number (Chr) and nucleotide position (start position of primer) in the Atlantic cod genome (version gadMor3). (for primer sequences see Johansen et al. 2018)

**Suppl. Table S2:** Comparison of otolith classifications between two experienced lab-technicians (Reader #1 and #2) in blind experiment. The same set of 297 otoliths were classified as CC or NEAC with high (types 1 or 5, resp.) or low (types 2 or 4) confidence. Differences in classifications between reader #1 and #2 occurred only for otoliths (78 in total) that were judged as uncertain by at least one.

|  |  |  | Reader #1 | | | |
| --- | --- | --- | --- | --- | --- | --- |
|  | Otolith type | | CC | | NEAC | |
|  |  |  | certain (1) | uncertain (2) | uncertain (4) | certain (5) |
| Reader #2 | CC | 1 | 48 | 6 | 5 | 0 |
|  |  | 2 | 9 | 5 | 11 | 4 |
|  | NEAC | 4 | 5 | 3 | 3 | 14 |
|  |  | 5 | 0 | 1 | 12 | 171 |


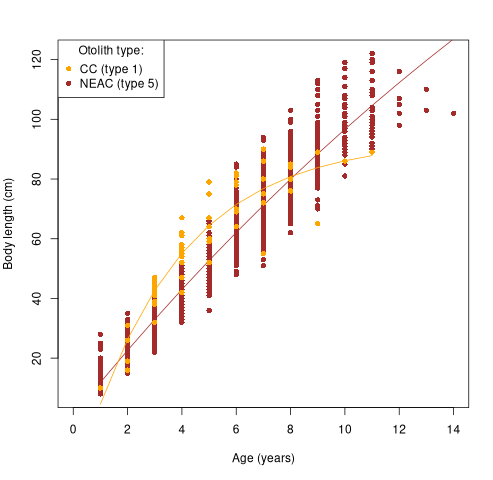


**Suppl. Figure S1.** Age and body length (dots) and estimates of von Bertalanffy growth function (lines) in cod classified as “CC” or “NEAC” based on otolith types (readings from surveys: NEAC (type 5), n=2546; CC (type 1), n=69).


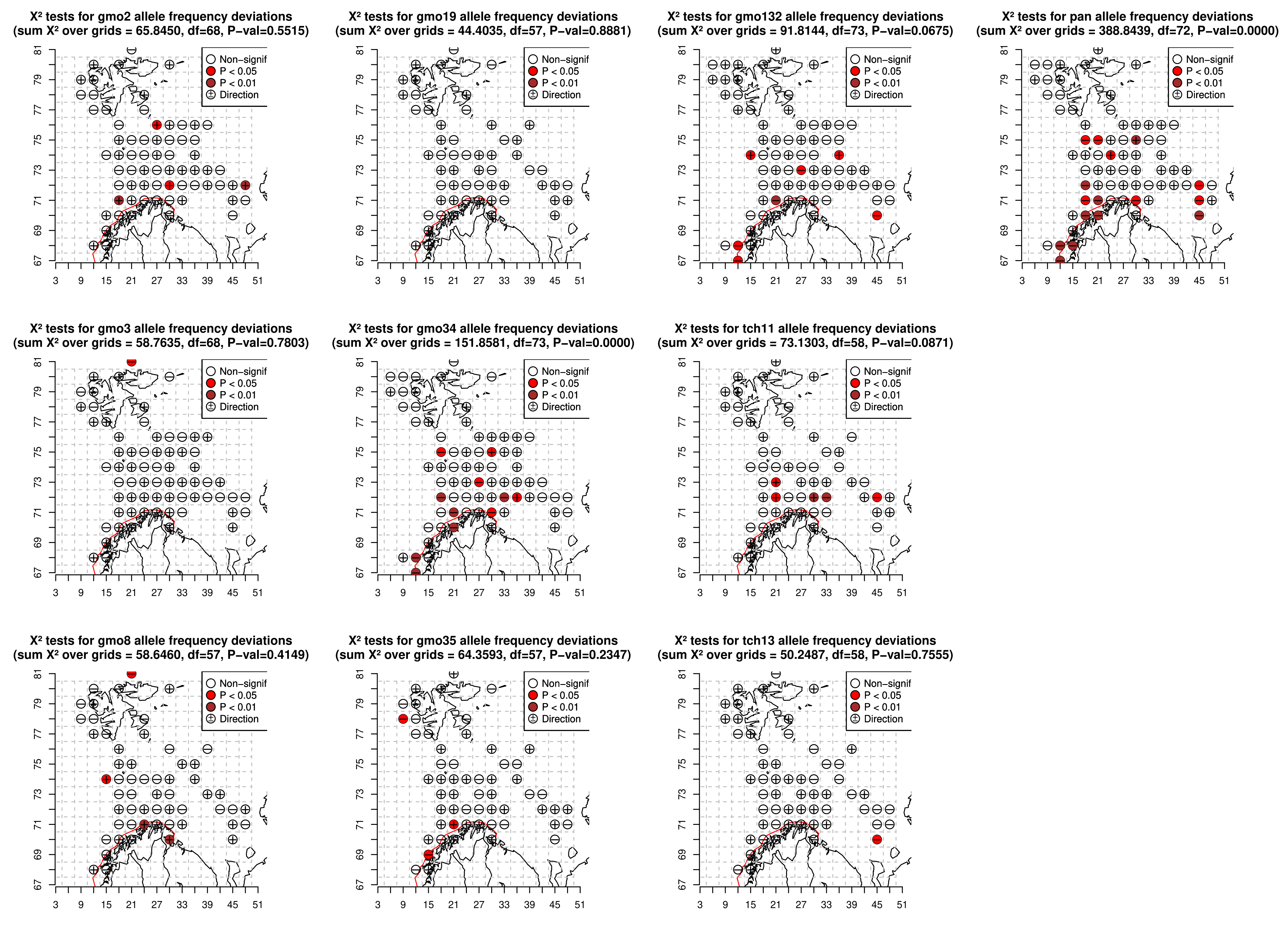


**Suppl. Figure S2.** Spatial tests for allele frequency deviations in 9 microsatellites and *Pan I* from the Barent Sea average frequency (based on 2793 cod caught off-shore from the red coastline). Samples are binned into cells of 1 degree latitude and 3 degrees longitude. For cells that do not overlap the red coastline, average allele frequencies (pooling minor alleles at microsatellite loci) were calculated and used in tests for allele frequency deviations. For each cell, observed allele frequencies were tested for goodness-of-fit to these averages using a chi-square test with df=1. The test result for each cell is coloured according to significance level (red for α < 0.05, brown for <0.01) and are not adjusted for multiple tests (cells). The direction of major allele frequency deviations from the mean are indicated by a sign (+/-) whether statistically significant or not. Multiciply of tests (cells) is implicitly accounted for in a global test, summing chi-square values over all cells, with details given in the title for each panel (locus).


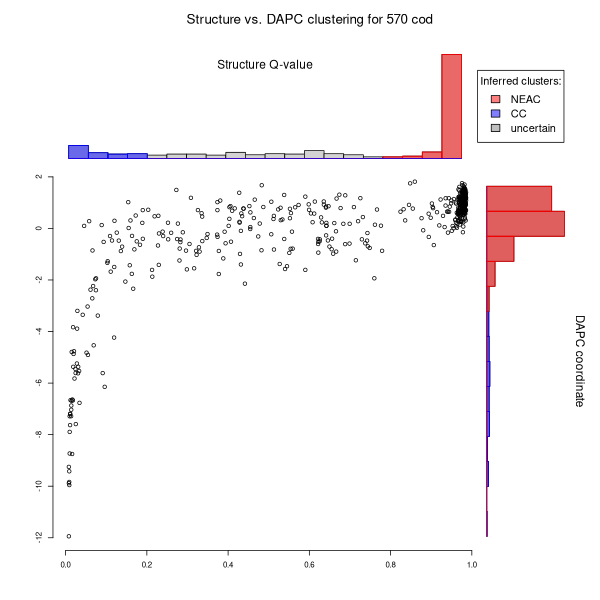


**Suppl. Figure S3.** Classification of 570 cod (open circles) based on 36 SNPs into K=2 groups, with STRUCTURE (horizontal axis) and DAPC (vertical axis). Marginal histograms represent the disribution of individual statistics (STRUCTURE q-values or DAPC coordinates) with bars colored according to inferred biological populations(NEAC: red; CC: blue). In STRUCTURE, individuals with intermediate q-values (between 0.2 and 0.8) were left unassigned (gray bars).


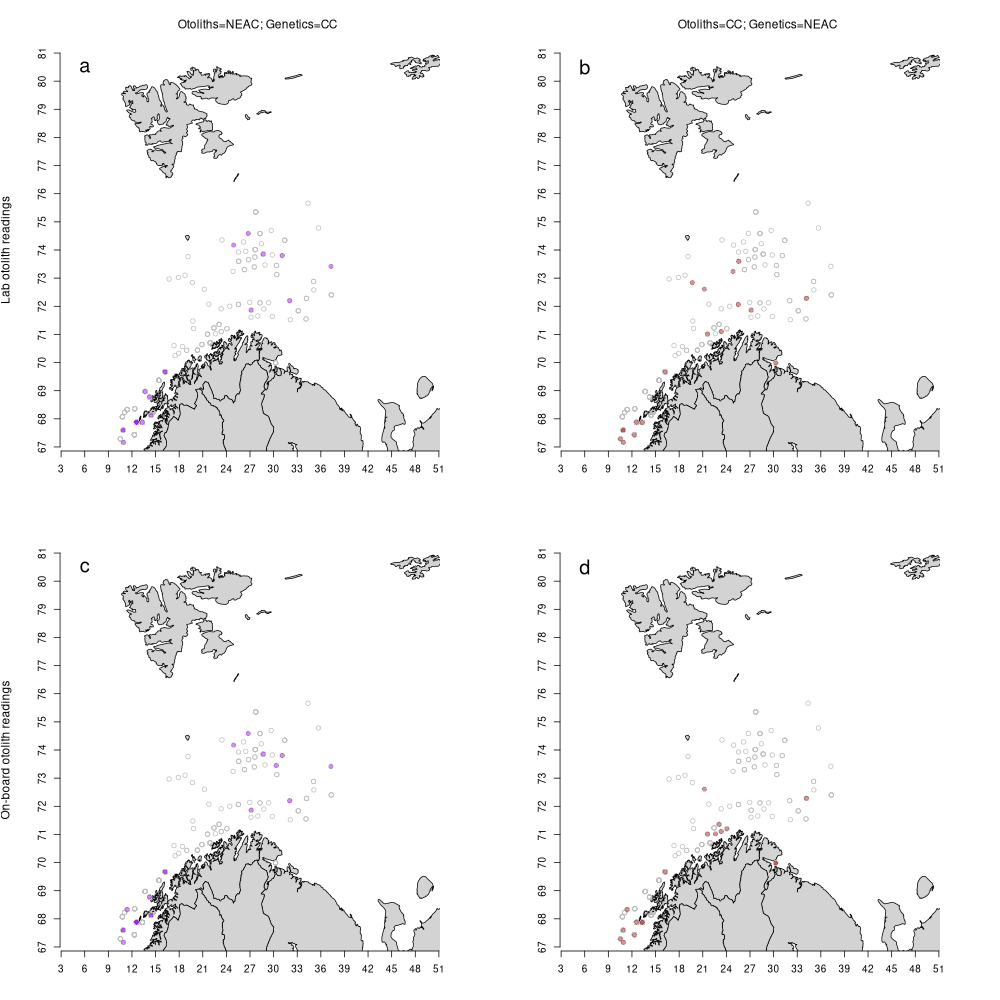


**Suppl. Figure S4**. Position of cod with mismatching ecotype classifications based on otolith vs. genetics (STRUCTURE K=2 classes, using 36 SNPs). **Left panels (a+c)**: Otolith type NEAC (type 5) but genetically CC. **Right panels (b+d)**: Otolith type CC (type 1) but genetically NEAC. **Top panels (a+b):** on-board otolith typing. **Bottom panels (c+d)**: lab census otolith typing. Colored circles: position of cod with otolith-genetics type conflicts (darker colours indicates >1 individual). Open circles: positions with no otolith-genetics type conflicts.
